# Surface Metrology Based on Scanning Conoscopic Holography for In Situ and In-Process Monitoring of Microtexture in Paintings

**DOI:** 10.3390/s22176637

**Published:** 2022-09-02

**Authors:** Claudia Daffara, Sara Mazzocato

**Affiliations:** Department of Computer Science, University of Verona, Strada le Grazie 15, 37134 Verona, Italy

**Keywords:** surface metrology, surface roughness, 3D optical profilometry, conoscopic holography, heritage science, painting treatment, laser cleaning

## Abstract

In the field of engineering, surface metrology is a valuable tool codified by international standards that enables the quantitative study of small-scale surface features. However, it is not recognized as a resource in the field of cultural heritage. Motivated by this fact, in this work, we demonstrate the use and the usefulness of surface metrology based on scanning conoscopic holography for monitoring treatments on the Venetian masterpiece by Tintoretto *St. Martial in Glory with the Saints Peter and Paul*. We carried out in situ and in-process monitoring of the painting microtexture during an experimental, innovative laser–chemical treatment, and we performed a statistical analysis based on ISO areal field parameters. A wide and in-band roughness analysis through the complementary use of amplitude, spatial, and hybrid parameters confirmed the noninvasive nature of the whole treatment on the painting surface topography, giving us the chance to review and critically discuss the use of these parameters in a real case in heritage science.

## 1. Introduction

### 1.1. Background and Motivation

In this work, we discuss the potentialities of surface metrology based on conoscopic holography sensors for monitoring treatments in ancient paintings and give a proof-of-concept on the notable masterpiece by Tintoretto *St. Martial in Glory with the Saints Peter and Paul* situated in Venice in the Church of San Marziale.

Surface metrology is applied in the field of engineering to understand the surface topography, namely the quantitative small-scale features beyond qualitative morphology, but it is not considered a useful tool in heritage science [1]. The main difficulty is that, while industrial surface metrology analyzes machined metal pieces with an expected surface pattern and known materials, each artwork is a unique piece composed by heterogeneous materials that have undergone aging and restoration processes over centuries. Without entering the debate regarding the meaning of setting international standards for artworks (e.g., ISO standards) [2], surface metrology techniques are useful for estimating micrometric changes in surface texture that help heritage scientists discriminate several characteristics of a painting or the processes it undergoes [3]. Moreover, optical techniques are available for noninvasive sampling of the surface microtexture and in-process metrology [4,5]; among them, conoscopic holography [6] was shown to be able to provide accurate surface datasets also on heterogeneous (diffusive, highly reflective, or polychrome) artworks [7,8].

To celebrate 500th anniversary of Tintoretto’s birth [9], this large oil painting on canvas was subjected to a complete restoration, during which an experimental, innovative laser–chemical treatment was performed by Brunetto et al [10]. The painting presented a degraded surface with a difficult situation, due to the oxidation of the pigmented varnish layer applied in old restorations, which was solved by the restorers with a multi-step cleaning procedure based on Er:YAG laser. The intervention was monitored by imaging and spectrometry techniques that provided information about the painting features and materials. In this context, we were called to perform a surface analysis with a customized scanning profilometer [7] based on conoscopic holography sensors in order to obtain data about the structure of the painting texture at microscale, complementary information not collected by the available, conventional in situ diagnostics.

The use of erbium lasers in cultural heritage has been recently reviewed in the literature [11], including the discussion of the physicochemical mechanism of the laser–matter interaction in paintings and the current approaches to monitoring [12,13]. Diagnostics of paintings cleaning are being carried out by multiple noninvasive techniques for inspecting materials and laser effects [14,15,16], e.g., spectral imaging and spectroscopy methods for probing materials, and interferometric methods such as optical coherence tomography (OCT) able to probe the layers stratigraphy. Optical profilometry based on conoscopic holography sensors was shown to be useful as a complementary technique for a qualitative inspection of the surface morphology in ancient paintings [17,18,19]. Surface data are rendered as raking light and fused with multispectral images [18], or used for holistic inspection of surface layers thickness [17,19] that are then measured by OCT.

As a matter of fact, despite the recognized potential of the technique, surface metrology for painting diagnostics is not being explored and we are motivated to bridge the gap.

The aim of this cross-disciplinary research is to test and validate a surface metrology workflow, based on conoscopic holography sensors and standard surface descriptors, for in-process monitoring of the microtexture in ancient paintings, toward a more general use in artwork diagnostics.

### 1.2. Paper Organization

In the above framework of an interdisciplinary research involving surface metrology in heritage science, which is not a routine diagnostic technique in the field, we first recall, in the Materials and Methods, the surface descriptors. We start from the definition and a review of the functional meaning to approach the possible use for painting analysis (Section 2.1). Then, we present the design of exemplar experiments, based on a real-world case study on Tintoretto, and the data acquisition in situ using scanning profilometry based on conoscopic holography sensors, giving the surface metrology workflow.

The results are discussed to address two aspects: one related to the surface metrology framework and the other one related to the specific conservation application, the laser treatment. Attention is paid to peculiar aspects of the heritage applications.

## 2. Materials and Methods

### 2.1. Surface Metrology Based on Areal Field Parameters

Surface metrology on paintings was implemented using areal field parameters [20], which were computed on the entire sampling area and were well-defined. Following the ISO 25178 standard [21], the general mathematical definitions for the continuous case are given here; the implementation for the discrete dataset was made using summations in Matlab environment [22]. Further details on areal surface texture parameters can be found in the specific literature (see the review [23]). A roadmap on challenges and open questions in surface topography measurement was given by [24].

The real surface of the painting can be represented as a 2D continuous function of height, which was sampled by the measurement at discrete points in a selected area. In the following equations, z(x,y) represents the centered height function with reference to the mean plane computed in the definition area *A*. In the experimental dataset, we have an array of surface heights $z_{ij}$ sampled in a spatial grid at intervals given by the scanning step of the profilometer device.

#### 2.1.1. Amplitude Parameters

The root mean square (RMS) roughness (Sq), defined as

(1) $$Sq=\sqrt{\frac{1}{A}\int \int_{A}z^{2}\left(x,y\right)dxdy}$$

was used as a descriptor of the mean height of the surface asperities. From a statistic point of view, it is the standard deviation of the probability density distribution of the heights, the amplitude distribution function (ADF).

The shape of the ADF is described by the skewness (Ssk) and the kurtosis (Sku), respectively, the third and the fourth statistical moments

(2) $$Ssk=\sqrt{\frac{1}{A}\frac{1}{Sq^{3}}\int \int_{A}z^{3}\left(x,y\right)dxdy}$$

(3) $$Sku=\sqrt{\frac{1}{A}\frac{1}{Sq^{4}}\int \int_{A}z^{4}\left(x,y\right)dxdy}\;.$$

The Ssk parameter measures the degree of asymmetry of the height distribution, that is, if the surface exhibits the bulk of materials above (Ssk<0) or below (Ssk>0) the mean plane. The Sku parameter represents the sharpness of the heights distribution, with the presence (Sku>3) or a lack (Sku<3) of inordinately high peaks/deep valleys. A surface with a symmetric, Gaussian height distribution has zero skewness and a kurtosis value of three.

The maximum height of the surface (Sz) was computed as the sum of the absolute values of the maximum peak height (Sp) and the maximum pit height (Sv) from the mean plane

(4) Sz=Sp+|Sv|.

From surface metrology literature [23], extreme parameters are being considered in relation to the surface damage, while the averaged parameters are related to functional aspects of the surface.

However, in cultural heritage applications, attention must be paid to the fact that an artifact is a complex object with a hand-processed surface, characterized by a non-homogeneous micro-geometry. Moreover, surface damages and modifications may be induced by various external factors, not controlled, related to the processed materials and interactions with the environment (microclimate), as well as to the human intervention (e.g., conservation treatments).

#### 2.1.2. Spatial Parameters

The power spectrum density (PSD) of a rough surface is defined as

(5) $$PSD\left(q_{x},q_{y}\right)=\frac{1}{{\left(2\pi \right)}^{2}}\int \int_{A}\langle z\left(x,y\right)z\left(0,0\right)\rangle e^{-i(q_{x}+q_{y})}dxdy\;,$$

where 〈⋯〉 is the ensemble average and $q_{x},q_{y}$ are wavevectors in the x and y directions ($q_{i}=2\pi /\lambda_{i}$, where $\lambda_{i}$ is the spatial wavelength). The PSD is a useful tool that allows for highlighting the contribution of different spatial wavelengths.

From the literature, the surface roughness power spectrum provides statistical information on the surface topography that carries insights about contact mechanics, friction, and adhesion, as reviewed in [25]. It is particularly effective in comparing surfaces measured with different instruments and techniques [26], i.e., with different spatial-frequency bandwidth limits. A review on the quantitative characterization of surface topography using spectral analysis is given in [27]. A PSD analysis of surface profiles can also be employed in the detection of and reduction in high-frequency measurement noise [28].

PSD-based techniques are thus expected to have an impact on the study of surface treatments in artwork conservation, involving multiscale processes, that are measured by multimodal techniques. The PSD function is interesting for controlling the performance of scanning microprofilometry in an out-of-laboratory environment, i.e., a museum, that is subjected to vibration.

In cultural heritage applications, the multiscale approach based on PSD has been recently demonstrated by the authors to compare the surfaces of an archaeological object and its 3D printed replica [29] and to validate the haptic fruition of artworks [30].

#### 2.1.3. Hybrid Parameters

The ISO standard provides two hybrid parameters, which combine amplitude and spatial information and are interrelated. The RMS gradient or slope (Sdq) is defined as

(6) $$Sdq=\sqrt{\frac{1}{A}\int \int_{A}\left[{\left(\frac{\partial z(x,y)}{\partial x}\right)}^{2}+{\left(\frac{\partial z(x,y)}{\partial y}\right)}^{2}\right]dxdy}\;,$$

and it can be used for inspecting surface anisotropy and cosmetic appearance [20]. Among the optical techniques, the conoscopic holography adopted in this study was shown to be effective for measuring steep slope surfaces [31].

The developed interfacial area ratio Sdr was calculated by summing the local area when following the surface curvature

(7) $$Sdr=\frac{1}{A}\left[\int \int_{A}\left(\sqrt{1+{\left(\frac{\partial z(x,y)}{\partial x}\right)}^{2}+{\left(\frac{\partial z(x,y)}{\partial y}\right)}^{2}}-1\right)dxdy\right]\;.$$

It can be used as a measure of the surface complexity when analyzing the steps of surface processing. A flat and smooth surface has an Sdr value of zero.

From the literature [3], hybrid parameters characterize the shapes of the structures of the painting, which influence the specular and diffuse rendering of the light on the artwork.

### 2.2. Design of the Validation Experiments: The Treatment of the Tintoretto Masterpiece

The experiments were performed on the altarpiece by Tintoretto *St. Martial in Glory*, situated in the Venetian Church of San Marziale. Beside the importance of the Tintoretto canvas as a historical masterpiece, it is an exemplary case study for our aims. Specifically, due to the oxidation of the old pigmented varnish layer and the presence of degraded products, the painting surface was subjected to an experimental multi-step cleaning treatment performed with Er:YAG laser (λ=2400 nm) in combination with different solvent mixtures [10].

In order to demonstrate the role of surface metrology for in situ and in-process painting monitoring, test areas were prepared ad hoc by the restorers for exemplary experiments with the microprofilometer. Different measurement sessions were carried out to analyze the condition of the microtexture of the painting during the laser and the chemical cleaning procedures.

**Figure 1:**
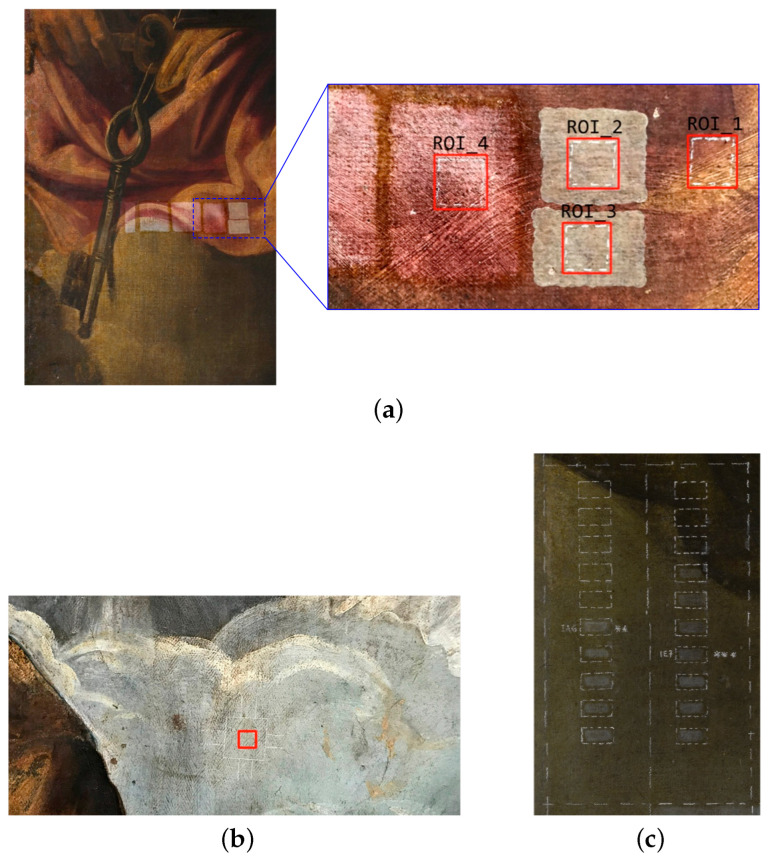
Pictures of the surface samples with the cleaning test detailed in Table 1. Experiment 1: (**a**) Multiple ROIs of red lake on St. Peter’s cloak with the treatment steps, original non-treated (ROI 1), laser (ROIs 2 and 3), laser–chemical (ROI 4). (**b**) Single ROI of azurite mixed with calcium carbonate, lead white, red ocher, and carbon black. Experiment 2: (**c**) ROIs labeled with stars treated with different solvent mixture at different concentrations.

1 *Multi-step treatment laser–chemical*
  The experiment analyzes the surface processed with the two-phase laser–chemical cleaning treatment. The different steps of the procedure are studied in a single region of interest (ROI) or in multiple ROIs, thus testing an “in-process” or an “off-line” diagnostics. Multiple ROI testing is necessary when performing the optimization of parameters (e.g., the laser pulse) for a single step.
  **Multiple ROIs** Profilometry is performed on different test areas of a red lake painted surface, in which the main steps are reproduced: a ROI is not treated, two ROIs are treated with the laser set at different pulse modes, and a ROI is treated with the laser combined with a specific chemical treatment.
    The typical practice, in which the restorer prepares various test areas of the treatment, for a performance analysis is carried out off-line (see Figure 1a).
  **SingleROI** Profilometry is performed on a region painted with azurite and lead white, which was pre-treated with solvents to remove most of the degraded upper layer (old varnish and candle soot), leaving residues [10]. The ROI is subjected to the whole laser–chemical cleaning treatment and the temporal evolution of the surface is monitored at different steps of the procedure.
    It is a controlled situation, in which an in-process diagnostics is performed in synergy with the restorer (see Figure 1b).
2 *Chemical treatment steps*
  The experiment analyzes the effects of the chemical treatments on the microsurface. In this case, different ROIs treated with different solvent mixtures are acquired.
  A typical situation is the quality control being performed off-line on the final surface subjected to different processing (see Figure 1c).

Table 1 summarizes the surface treatment samples for the experiments (further details in [10]).

**Table 1:** Description of the surface treatment samples.

Experiments	ROI	Laser Er:YAG	Solvent
1. Multi-step treatment:			
a. Multiple ROIs, red lake	A1	/	/
	A2	Very Short pulse ^1^	/
	A3	Short pulse ^2^	/
	A4	Short pulse	isooctane 80% ethyl alcohol 20%
b. Single ROI, azurite	T0,T1,T2	Short pulse	isooctane 80% ethyl alcohol 20%
2. Chemical treatment steps	1, …, 10	/	10%, …, 100% ethyl alcohol
	1, …, 10	/	10%, …, 100% acetone

^1^ In very short pulse mode (150 μs), with 150 mJ nominal energy and a spot diameter of 4.5 mm, 5 mm and 6 mm at 10 Hz and 15 Hz, the fluence was from 0.32 J/cm^2^ to 0.65 J/cm^2^. ^2^ In short pulse mode (250 μs–300 μs), with 150 mJ to 200 mJ nominal energy and a spot diameter of 5.5 mm, 6 mm and 7 mm at 10 Hz, 15 Hz and 20 Hz, the fluence was from 0.38 J/cm^2^ to 0.55 J/cm^2^.

### 2.3. Microprofilometry Based on Conoscopic Holography Sensors for In Situ Measurements of Paintings

In order to effectively acquire the surface texture of polychrome paintings in situ, we optimized the setup of a portable microprofilometer based on interchangeable single-point, conoscopic holography depth sensors, which was implemented by our group and first described in [7]. A comprehensive description of the project developed for surface microprofilometry on artworks based on scanning conoscopic holography can be found in [2].

**Figure 2:**
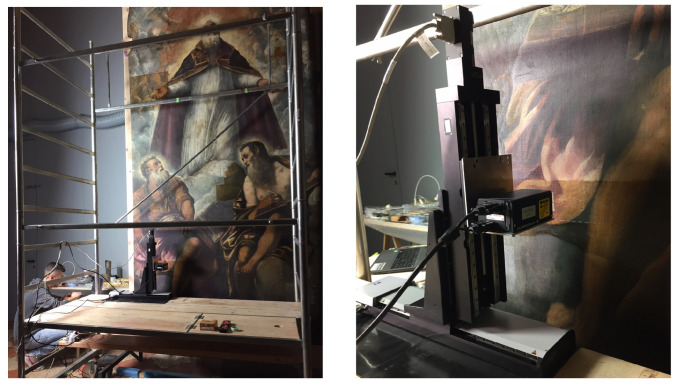
Scanning profilometry on the Tintoretto masterpiece *Saint Martial in Glory*, altarpiece from the church of San Marziale in Venice (Italy). The conoscopic holography sensor is mounted on micrometric stages to scan the painting in the vertical plane at safe working distance.

The measurements were carried out in situ, in the Venetian church, during the restoration of the painting (see Figure 2).

The system provides a high resolution in both the vertical (down to the order of sub-micrometers) and lateral (down to the order of micrometers) directions. Two orthogonally mounted linear stages move the optical probe to scan the painting plane vertically, at fixed working distance, allowing for micrometric measurement of the surface heights, as depicted in the setup in Figure 2. The painting surface is sampled as a set of horizontal profiles. The motion system covers a scan area of 30 cm × 30 cm with a minimum incremental step of 0.1 μm. The scanning measurement is non-contact and noninvasive for the pictorial matter, the single-point conoscopic holography depth sensor uses a class 2 laser source (<1 mW) of 655 nm operating wavelength.

Performing micrometer measurements in an out-of-lab environment is challenging, specifically in Venice, where ground vibrations are an unavoidable factor. Before carrying out the in situ measurements, we carried out optimization tests for the best trade-off between parameters, e.g., scanning velocity and probe-lens coupling. The optimized setup involved the use of the 100 mm lens with the device mounted on a sorbothane damping base and a scan velocity set at 5 mm/s. The scanning of the Tintoretto canvas was performed in continuous mode with the probe in pulse-triggering and a sampling spatial step of 50 μm in both the scan (horizontal) and subscan (vertical) directions.

The depth sensor parameters are summarized in Table 2. A depth repeatability of 1.5 μm was estimated on the ancient painting. Spectrometry testing allowed us to investigate the laser response on the polychrome paint layer, assisting in finding the finest settings [7]. The laser performance was then tweaked before starting each measurement session, finding the best options in terms of power and frequency. Then, the quality was evaluated using the signal-to-noise ratio, the total signal collected, and the correct working distance based on lens-probe coupling [32].

### 2.4. The Surface Metrology Workflow

**Figure 3:**
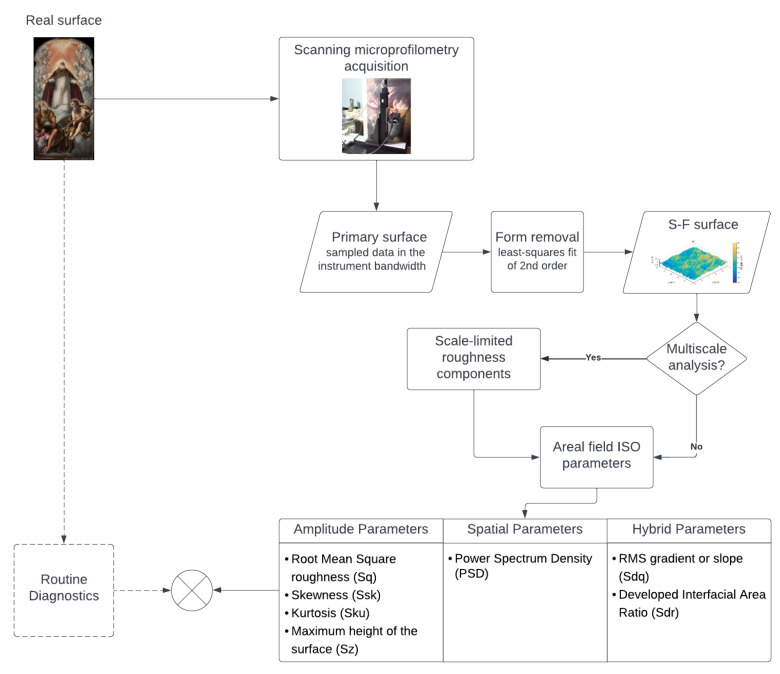
Workflow diagram in the general framework of metrology in cultural heritage. Algorithms are implemented in MatLab environment, starting from the open-source SCOUT toolbox [35]. Scale-limited roughness components separation is not carried out in the present work, but an example of multiscale analysis performed by the authors can be found in [8].

Figure 3 describes the workflow of this work within the general framework of involving surface metrology in heritage science.

The real surface was digitized in the instrument bandwidth, obtaining the so-called primary surface. The form of each surface acquired was then removed by a least-squares fit of second-order polynomial. In this specific case, the support of the painting was a canvas, and a surface analysis was performed on a near planar patch [33]. Thus, removing a second-order polynomial as a general form is, in our experience, the preferable approach in this case study. It is also worth noting that topography filtering that employs the measurement length as a cut-off can be used, e.g., robust Gaussian or Legendre polynomials [34]. The metrology parameters are then calculated in the scale-limited surface S-F in the bandwidth set by the x/y resolution of the scanning profilometer and the measurement x/y length.

## 3. Results

### 3.1. Multi-Step Treatment Laser–Chemical

The evolution of the microsurface during the two-step process (laser + chemical) is analyzed in a single and in separate ROIs. In addition, the multiple ROI experiment allowed for testing the surface processed with the Er:YAG laser set in different modes.

**Figure 4:**
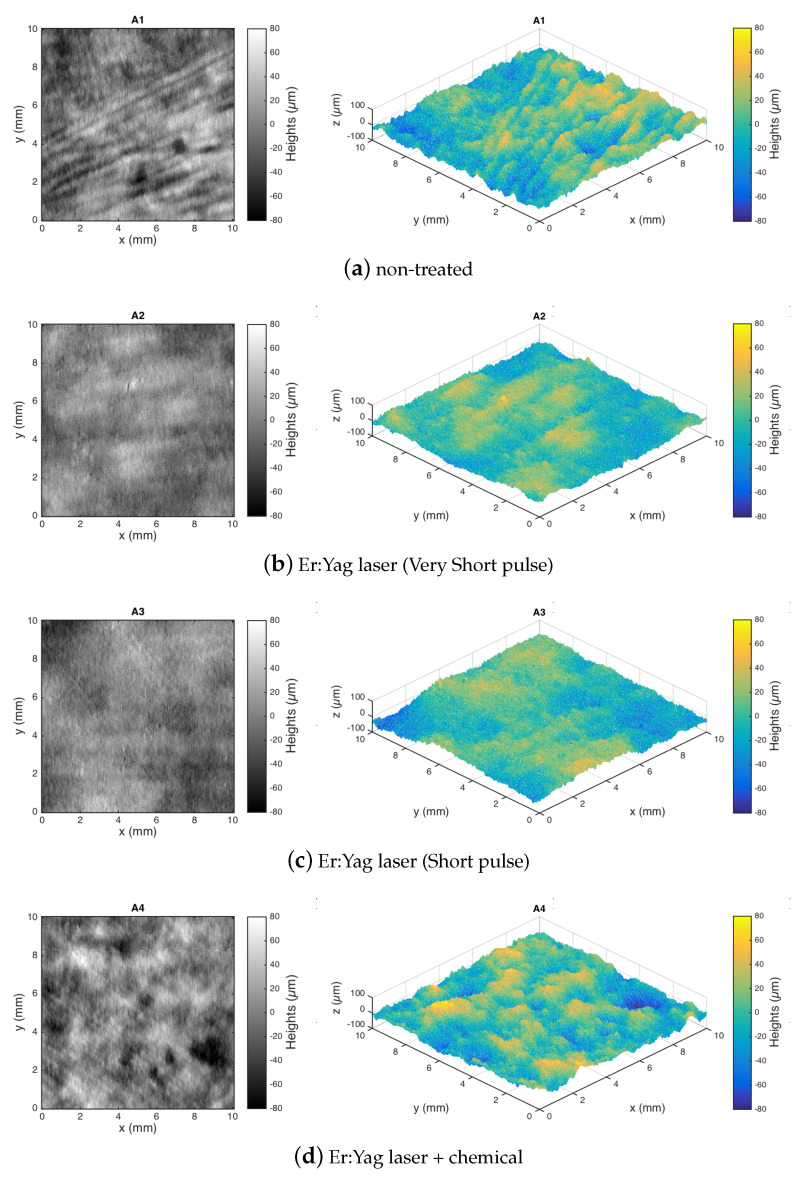
Experiment 1-dataset (**a**). Different red lake ROIs with different surface processing: surface data plotted as intensity maps and 3D visualization.

**Figure 5:**
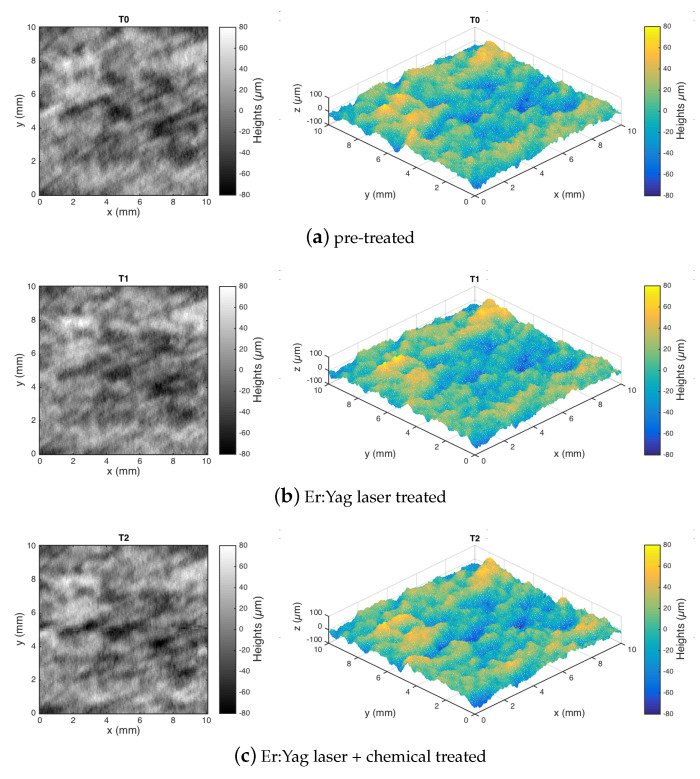
Experiment 1-dataset (**b**). Evolution of a single azurite ROI at different surface processing steps: surface data plotted as intensity maps and 3D visualization.

Following the workflow (Figure 3), the two- and the three-dimensional topography maps shown in Figure 4 and Figure 5 were obtained from the surface heights data acquired by the microprofilometer after the form removal (least-squares fit of second-order polynomial) and represent the leveled surface texture. The threshold for the outlier was set to three-sigma; the settings of the conoscopic holography sensor and scanning parameters were effective in sampling the diffusive, polychrome painting surface. Surface metrology was thus carried out on this dataset.

From a preliminary, qualitative inspection of the heights map, the surface before and after the laser–chemical treatment is similar in its appearance and micro-geometry. The surface topography appears random in its micro-structure. No defects, deformations, or pattern left by the laser processing and the chemical removal were visually observable in the final surface. This is clearly evident in Figure 5 (same ROI), while in Figure 4 (separate ROIs), the different local features, like the painting strokes, are visible. The smoothing of the surface texture in the intermediate laser processing step, visible in the red lake region (Figure 4), is the effect of the laser on the layer of pigmented varnish, not present in the pre-treated azurite region (Figure 5).

**Figure 6:**
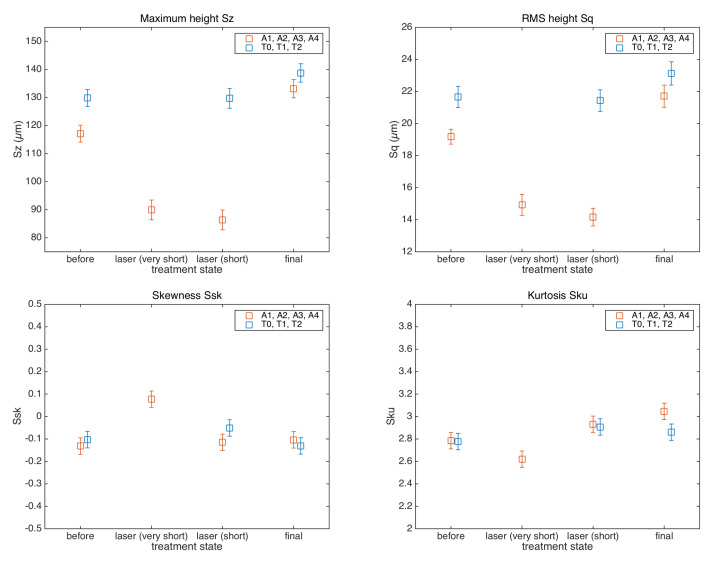
Amplitude roughness parameters of the painting surface at the different processing steps: before the treatment, intermediate (laser), and final (laser+chemical). The plot combines the results of the experiments in the azurite (blue markers) and red lake (red markers), with the cleaning treatments applied in multiple and single ROI (see Table 1).

Quantitative analysis was then performed using the areal field parameters given in Section 2.1. The amplitude parameters Sz, Sq, Ssk, and Sku were computed for each sample and compared vs. the treatment state in Figure 6.

First, the maximum height Sz was analyzed as an indicator of possible surface damage. As mentioned before, the values of some roughness parameters may clearly depend on the position of the ROI across the non-homogeneous painted surface; here, the observed variations are also in relation to different painting features such as the brushstrokes. The non-treated surfaces were characterized by Sz values of the same order, and the values in the final treated areas showed that the surface at microscale is preserved at this level, thus suggesting that the laser–chemical cleaning procedure can be considered noninvasive. Indeed, no formation of anomalous peaks or pits is observable in the surface topography (Figure 4). The smoothing of the red lake surface in the laser steps, observed above in the surface morphology, corresponds to a ΔSz∼30 μm, for the two laser settings.

As further confirmation of noninvasive surface processing, the RMS roughness Sq was observed to have a similar value in the non-treated surface and in the one subjected to the entire two-step treatment. It is interesting that the same result was observed when monitoring the single ROI of azurite, ΔSq=(1.5±1) μm, and when monitoring the surface in separate ROIs of red lake, ΔSq=(2.5±0.8) μm. Again, a lower Sq roughness was observed in the red lake due to the softening effect of the laser on the varnish layer, which was removed by the chemical process. As expected, the effect of the cleaning treatment was a sharper final texture.

We do not observe differences in the samples treated with different laser settings analyzing the amplitude parameters.

**Figure 7:**
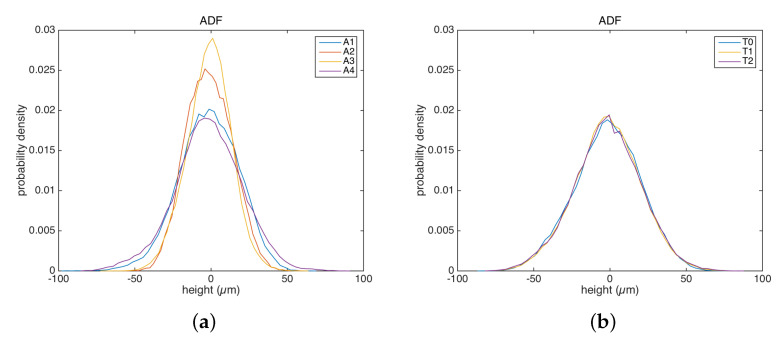
Probability density function of the heights: (**a**) multiple ROIs of red lake. (**b**) Single ROI of azurite.

The averaged parameters Sq, Ssk, and Sku may be further analyzed concerning the surface functioning. A conservation of the Sq micro-roughness is important in relation to the glossiness of the painting surface. Sq was found to have similar values on different areas of different pigments. Regarding the higher moments, we observed, in general, that the painting surface has a symmetrical ADF (Figure 7) with a nearly null skewness and a kurtosis around the normal value, before and after the treatments. The surface was not characterized by pores or cracks, and the distribution of peak and valleys was almost symmetric with respect to the mean plane. We did not observe strong variations in the Ssk and Sku parameters that would have indicated a response of the surface to an invasive cleaning treatment.

**Figure 8:**
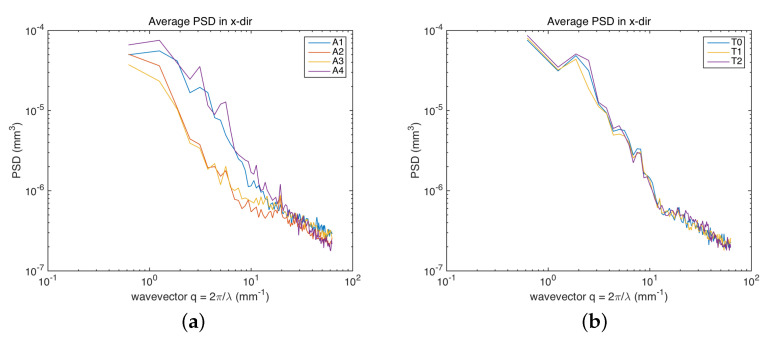
Average PSD along the scan direction: (**a**) multiple ROIs of red lake. (**b**) Single ROI of azurite.

It is interesting to compute the power spectrum of the surface heights, from which the contribution to the overall roughness at different frequency bands can be inspected. Figure 8 represents the averaged one-dimensional PSD in the horizontal (scan) direction. The effect of the laser cleaning is particularly evident in the red lake (Figure 8a): the PSDs computed for the surfaces not treated (A1) and treated (A2,A3) showed a decrease in the mid-low frequencies (from a spatial wavelength around 3 mm) due to the softening effect of the laser on the varnish layer. Then, the chemical treatment restored a surface (A4) similar to the initial one. In the case of the azurite (Figure 8b), most of the varnish layer was removed before the treatment, and hence, the PSDs were similar for the surfaces T0, T1, and T2. The in-band texture component analysis based on PSD thus supports and completes the results obtained above, where the global roughness parameters were computed, further confirming the noninvasive nature of the overall treatment.

Again, we did not observe a clear difference in the samples treated with a different laser settings using spatial parameters. In the face of the same Sq value, it is interesting to observe a weak modification of the roughness signal around a spatial wavelength of 500 μm, increasing for the A3 sample (short pulse) with respect to A2 (very short pulse).

### 3.2. Chemical Treatment Steps

**Figure 9:**
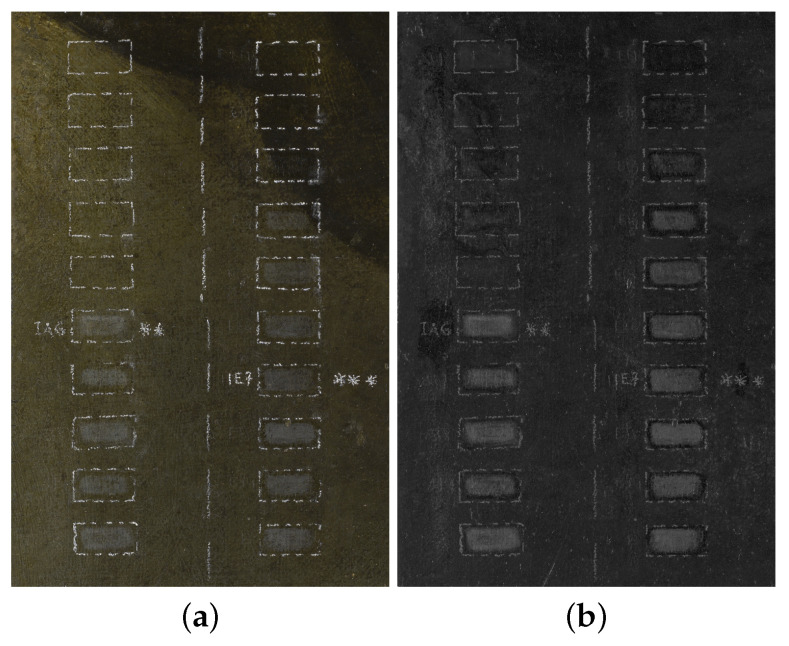
Technical photography: (**a**) VIS. (**b**) UV reflected. The ROIs were treated with solvent mixtures isooctane-acetone (left column) and isooctane-ethanol (right column), with the concentration of acetone and of ethanol increased at a step of 10% (top to bottom). ROIs labeled with stars represent two particular mixtures chosen by the restorer.

This experiment explored the effects of the chemical solvent cleaning on the painting surface. As reported in the recent review [36], this is a challenging topic that requires a strong collaboration between scientists and conservators. Figure 9 shows the experimental area with the ROIs treated with two binary mixtures of isooctane–acetone and of isooctane–ethanol at different concentrations. Technical photography in the UV range, both in luminescence and reflectance mode, was performed and jointly analyzed with surface microprofilometry, in order to allow for the monitoring of the cleaning action of the solvents.

As in the previous case, the form (least-squares fit of second-order polynomial) was removed from the surface height data acquired by the microprofilometer, with the outlier threshold set to three-sigma.

**Figure 10:**
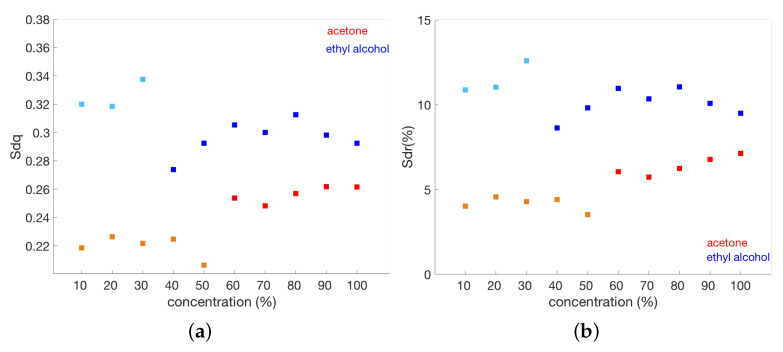
Variation in the hybrid parameters with solvent concentration: (**a**) RMS gradient Sdq. (**b**) Developed interfacial area ratio Sdr. Lighter colors represent the solvent concentrations before they become effective (based on UV imaging).

The solvent mixture became stronger with increasing concentrations of ethanol or acetone, but the first analysis concerning the variation in the RMS roughness parameter with the solvent action did not suggest a dependence. Thus, in order to investigate if there is a combined effect of texture spacing and texture amplitudes that differentiates the surfaces with similar average roughness, we computed the hybrid parameters RMS gradient Sdq and the developed interfacial area ratio Sdr. These parameters allowed us to characterize the structure of the surface, which influences the reflection of the light and, hence, the final visible aspect of the painting. As can be seen in Figure 10, when surface variations become evident in the UV response (see Figure 9b), there are changes in both the Sdq and Sdr parameters. The UV rays interact with the outermost surface layer, and hence, they are sensitive to what is or is not removed. At 40% of concentration of ethyl alcohol and 60% of acetone, there is a clear change in both the imaging results and the calculated parameters.

**Figure 11:**
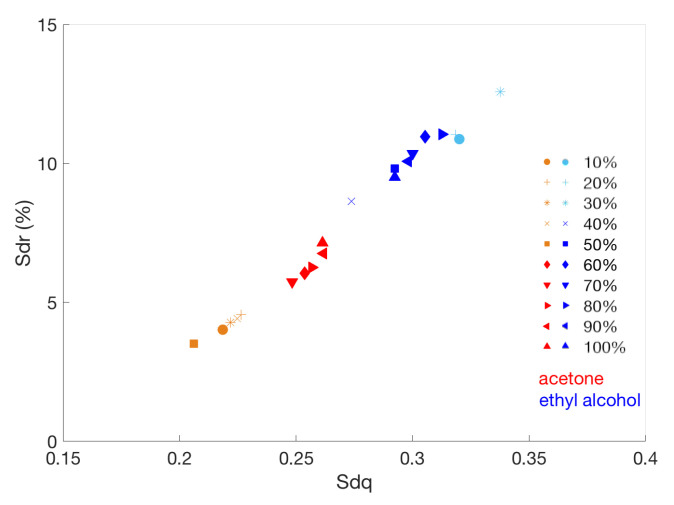
Interrelation between RMS gradient and developed interfacial area ratio for the different chemical cleanings. The different symbols represent the different concentrations of acetone (red-orange) and ethyl alcohol (blue-light blue).

As expected [37], the Sdq and Sdr parameters are interrelated (see Figure 11). Interestingly, there is a well-defined correlation of the overall cleaning process (both acetone- and ethanol-based) with the two different treatments, having their own range of specific values in the Sdq-Sdr space.

In the case of acetone-based cleaning, there is a clustering of values into two different groups before and after the effect of the solvent becomes evident, whereas this is less evident in the case of ethanol-based cleaning. As reported in the literature [23], the Sdr and Sdq parameters are related to the micro-geometry of the surface: greater Sdr means more complex micro-geometrical features while greater Sdq means that micro-asperities or peaks have a steeper or sharper slope. In this case, as both solvents become effective, the surfaces tend to show closer values in the Sdq-Sdr space.

## 4. Discussion

In addition to discussing the surface metrology workflow, the results obtained in the Tintoretto case study open up further consideration of various aspects. Within the main goal of demonstrating the possible role of surface metrology in heritage science, it is interesting to point out the position of the technique with respect to the routine diagnostics. Furthermore, it is discussed which painting surface features are analyzed in the proposed workflow.

### 4.1. Surface Metrology in Heritage Science

Above, we computed a quantitative analysis of the painting surface structure following a surface metrology workflow, from surface sampling to roughness analysis. In particular, we specifically explored the meaning of the ISO areal field parameters in the context of cultural heritage that represents a novel application field. In the face of this analysis, there are important heritage applications in which the analysis of surface topography in artworks is desirable: monitoring the cleaning treatments is an exemplary case.

The scanning profilometer based on conoscopic holography sensors [7] has proven effective in collecting meaningful data also in out-of-lab, real environments, thus enabling in situ and in-process painting monitoring. The triggered acquisition in continuous scanning at a resolution of 50 μm with a depth repeatability of 1.5 μm allowed us to sample the painting surface features and the calculation of the roughness parameters.

Scanning conoscopic holography is a recognized tool in painting diagnostics; however, as reviewed in the Introduction, it is mainly used for inspecting surface morphology or for single-parameter roughness estimations (usually Sq) but not within a surface metrology framework. The technique was specifically applied to layer thickness estimation in erbium laser ablation, mainly by height map subtraction [17], however recognizing the impossibility of aligning the range map before/after the treatment and preferring the OCT technique [19].

To innovate the field, the results in this study have demonstrated the combined use of the roughness parameters (amplitude, spatial, and hybrid) for characterizing the surface topography of the Tintoretto painting subjected to laser and chemical treatments, which is consistent and goes beyond the visual inspection of the surface map. The proof-of-concept experiment was given both in separate ROIs than in a single ROI, following its time evolution by spatially registering the regions down to single pixel (50 μm), as described in our previous work [30].

It is suggested that the maximum height Sz be used as a first indicator of possible damaged regions where anomalous peaks or holes are present. Higher moments are also useful to this purpose: in general, we have observed that, in the sampled bandwidth, the painting surfaces have a symmetrical ADF with a nearly null skewness Ssk and a kurtosis Sku around the normal value, implying a surface not characterized by pores or cracks with a distribution of peaks and valleys almost symmetrical. The RMS roughness Sq is used as descriptor of the mean height of the surface asperities when comparing the surfaces. In this case, the non-treated surface and the one subjected to the laser and chemical treatments have similar Sq values, supporting the noninvasive nature of the overall treatment.

The results have shown that, in heritage applications, the power spectrum PSD is an interesting tool as it allows us to investigate the roughness at different spatial frequency bands, thus enabling us to investigate the effects of multi-step treatments. The softening effect of the laser and the return of a surface very similar to the non-treated one after chemical removal were evident. Such an observation was deemed crucial for the conservators, confirming that their experimental cleaning treatment based on Er:Yag laser [10] was not invasive.

Hybrid parameters such as Sdr and Sdq that combine amplitude and spatial information can be useful when the other parameters are not very informative. Sdq gives an idea of how micro-asperities tilt sharper, while Sdr deals with overall complexity of the surface. Here, as a proof-of-concept experiment, we used these two parameters and their interrelation to monitor the effects of the different solvent concentrations on the painting surface during the chemical cleaning tests. Here, for example, the use of the single roughness parameter Sq did not provide results.

The surface metrology workflow (Figure 3) included the step of multiscale roughness analysis, which was not performed in this work as it would have required different hypotheses, namely, the sampling of surfaces in a larger spatial bandwidth. The limit of the 50 μm spatial step was imposed by the device setup in extreme conditions, while the *x*/*y* lengths (order of centimeters) were the size of the treatment tests made by the restorers. Multiscale analyses were previously demonstrated by the authors in the study of metal artworks treatments [8], using the scanning profilometer on optical table down to a spatial resolution of 5 μm and prepared mock ups. The application to paintings will be explored in our next work. However, an interpretation of waviness surface features in genuine artworks is difficult.

### 4.2. Average Roughness or Brushstrokes?

The main difficulty in cultural heritage applications is that ancient artworks are very complex objects, with the materials subjected to unknown processes across centuries. In this work, the surface samples are treated as cloud of points and a statistical analysis is performed using the areal field parameters. The surface of the Tintoretto painting, before or after the laser–chemical cleaning, is considered (locally) a stochastic-dominated surface and it is analyzed in term of average surface descriptors in small ROIs (order of centimeter). At the larger scale, an ancient painting, which is a hand-made artistic object with its complex and unique conservation history, can be likely treated as a stochastic-deterministic surface, on which advanced feature-based analysis [38] of topography could be tested, e.g., to study brushstroke pattern as an artist fingerprint [3]. To this regard, we point out that the possible pattern left by the manual cleaning treatment (i.e., by hand-held laser probe or solvent swabbing) across the painting was out of the aim of the present study and that the experimental test areas were prepared and supposed to be representative of the average painting surface.

### 4.3. Microprofilometry as Complementary Diagnostic Technique

**Figure 12:**
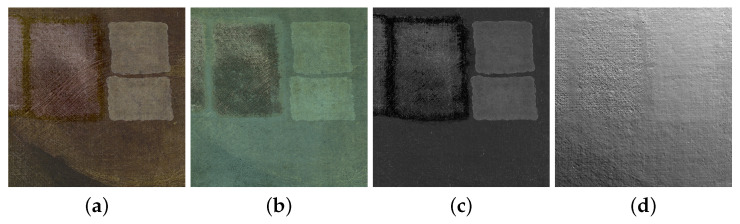
Technical photography on Tintoretto detail (experiment 1): (**a**) VIS. (**b**) UV-induced VIS fluorescence. (**c**) UV reflected. (**d**) RTI.

Finally, it is interesting to discuss the position of surface profilometry and roughness analysis with respect to the conventional techniques that are currently being used for in situ monitoring the cleaning treatment in paintings. In particular, among the routine diagnostics carried out by the conservators, an important role is played by the optical imaging techniques that allow for a full-field analysis with accessible equipment [10]. Figure 12 refers to the exemplary Tintoretto case study.

Concerning the painting surface morphology, raking light imaging or the more advanced reflectance transformation imaging (RTI) can be applied for an overall inspection of the surface micro-geometry, i.e., surface asperities and local features, after which a quantitative measurement with profilometry on selected areas is desirable. RTI is estimated to detect morphological changes and damage (e.g., holes and abrasion) in paintings down to 0.3 mm [39]; the microprofilometry validated here on Tintoretto provides topography maps at microscale and an average roughness estimation of ∼20 μm.

It is important to point out that, concerning the painting surface materials, the efficiency of the cleaning treatment with respect to the removal of the outermost layers is supervised in the VIS and UV images, while the profilometry provides the complementary analysis of the surface structure. The joint use of imaging and profilometry is being applied in painting diagnostics, showing that superimposition of 2D and 3D data can assist conservators [18]. A recent paper by Borg et al. [40] explored the joint use of pointwise spectroscopy and profilometry on a canvas mockup. The results obtained in our work show that profilometry supported by quantitave analysis within a surface metrology workflow (ISO standard) is consistent and effective in monitoring the laser and chemical treatment on ancient canvas in situ. This specific and difficult application emphasizes a general potential for artwork diagnostics.

## 5. Conclusions

In this research, the micro-geometrical features and related 3D surface texture parameters, measured with scanning conoscopic holography, were analyzed for in situ and in-process monitoring the cleaning treatment on a genuine canvas painting by Tintoretto, thus discussing a workflow for surface metrology in heritage science.

Microprofilometry of the painting was performed by non-contact optical method in an out-of-lab environment, specifically inside a Venetian Church, where external influences were unavoidable and were combined with intrinsic internal vibrations of the instrument, mainly due to the motorized stages. In this work, we have shown that the scanning conoscopic holography method is robust with respect to internal and external vibrations thanks to an optimized setup that required the use of a sorbothane damping support, the conoscopic depth sensor coupled to a 100 mm lens, and a 50 μm spatial step with a slow scanning using a high-precision stage system. A depth repeatability of 1.5 μm was estimated on the ancient painting.

We worked in synergy with the restorer who prepared ad hoc test areas for proof-of-concept experiments on surface metrology for painting diagnostics. The surfaces acquired in the measurement bandwidth were shown to be suitable in order to perform wide and in-band roughness analysis that allowed for a twofold discussion: on the one hand, the possible correlation with the performance of the laser and chemical surface processing, and on the other hand, practical use of the ISO areal field parameters in the heritage field, starting from the meaning and commonly accepted uses reviewed in engineering applications. As a result, a complementary use of amplitude, spatial, and hybrid parameters allowed us to assess the noninvasive character of the restoration treatment, a multi-phase procedure based on erbium laser and chemical steps, to the extent of which the average microscale surface amplitudes are preserved.

The study has demonstrated that the use of surface metrology techniques with conoscopy holography datasets enables the quantitative analysis of the surface topography in ancient paintings, i.e., beyond the widespread practice of a qualitative inspection of surface morphology or based on a single roughness parameter (usually Sq). Even if the definition of standard guidelines is generally not possible for ancient artworks, being unique and complex manufacts, the proof-of-concept carried out on Tintoretto clearly show the potential of the approach, thus calling scientists toward further cross-disciplinary research in order to bridge the gap between consolidated engineering metrology and unexplored heritage science applications.

## Figures and Tables

**Table 2:** Characteristic parameters of the conoscopic sensor ConoPoint-3 coupled to the 100 mm lens.

Stand-Off Distance	Working Range	Accuracy ^1^	Laser Spot	Repeatability ^2^
95 mm	35 mm	15 μm	63 μm	1.5 μm

^1^ Depth accuracy, defined as difference between two flat surfaces measured compared with the nominal value. ^2^ Depth repeatability, estimated as the standard deviation of 10,000 static measurements performed on a representative painting sample.

## Data Availability

The data acquired on artworks are not available for free access.

## Abbreviations

The following abbreviations are used in this manuscript:

Er:YAG	Erbium-Doped Yttrium Aluminium Garnet
OCT	Optical Coherence Tomography
RMS	Root Mean Square
ADF	Amplitude Distribution Function
PSD	Power Spectrum Density
ROI	Region of Interest
RTI	Reflectance Transformation Imaging
